# Key considerations for child and adolescent MRI data collection

**DOI:** 10.3389/fnimg.2022.981947

**Published:** 2022-09-12

**Authors:** Brittany R. Davis, AnnaCarolina Garza, Jessica A. Church

**Affiliations:** Department of Psychology, The University of Texas at Austin, Austin, TX, United States

**Keywords:** fMRI, brain, adolescence, development, diversity and inclusion, mental health, methods

## Abstract

Cognitive neuroimaging researchers’ ability to infer accurate statistical conclusions from neuroimaging depends greatly on the quality of the data analyzed. This need for quality control is never more evident than when conducting neuroimaging studies with children and adolescents. Developmental neuroimaging requires patience, flexibility, adaptability, extra time, and effort. It also provides us a unique, non-invasive way to understand the development of cognitive processes, individual differences, and the changing relations between brain and behavior over the lifespan. In this discussion, we focus on collecting magnetic resonance imaging (MRI) data, as it is one of the more complex protocols used with children and youth. Through our extensive experience collecting MRI datasets with children and families, as well as a review of current best practices, we will cover three main topics to help neuroimaging researchers collect high-quality datasets. First, we review key recruitment and retention techniques, and note the importance for consistency and inclusion across groups. Second, we discuss ways to reduce scan anxiety for families and ways to increase scan success by describing the pre-screening process, use of a scanner simulator, and the need to focus on participant and family comfort. Finally, we outline several important design considerations in developmental neuroimaging such as asking a developmentally appropriate question, minimizing data loss, and the applicability of public datasets. Altogether, we hope this article serves as a useful tool for those wishing to enter or learn more about developmental cognitive neuroscience.

## Introduction

Studies on child brain development capture the interest of the media, educators, policy makers, and parents alike. What happens in the brain when a child reads (e.g., Church et al., 2021)? How does the brain change when a teenager is engaging with social media posts (e.g., Sherman et al., 2016)? What parts of the brain expand or thin over development (e.g., Mills et al., 2021)? Developmental cognitive neuroscience, or the study of the human brain over infancy, childhood, and adolescence, is a rapidly growing field that contributes to our understanding of biological maturation as well as cognitive development. Developmental cognitive neuroscientists are investigating a wide range of questions about child health and cognition, starting even before birth (e.g., Dubois et al., 2014; Turk et al., 2019), and covering the whole pediatric lifespan (e.g., Jernigan et al., 2016; Nketia et al., 2021). Addition of a developmental data collection to a research question can greatly inform understanding of the plasticity and trajectory of a cognitive process, as well as inform how it can go awry at different points in the lifespan. This article seeks to offer some practical tips for researchers wishing to add developmental neuroimaging studies to their protocols, or for those simply wishing to better understand this growing and dynamic field.

Developmental cognitive neuroscientists currently have multiple non-invasive tools that provide unprecedented access to the child brain’s structure and function [e.g., functional near-infrared spectroscopy (fNIRS), electroencephalography (EEG), and magnetic resonance imaging (MRI)]. In this discussion, we focus specifically on MRI collection and its related data types [task- or rest-based functional MRI (fMRI), structural MRI, and diffusion-weighted imaging (DWI)], as MRI is one of the more complex non-invasive neuroimaging protocols currently used with children and youth.

When neuroimaging scientists research the developing brain’s *structure* with MRI, they are typically measuring how aspects of its size, folding, or anatomical connections differ over age, condition, or between different groups (e.g., those with and without a disorder of interest) (Lerch et al., 2017). In *diffusion* neuroimaging studies, researchers are measuring the movement of water molecules through different tissues to visualize white matter anatomy (Qiu et al., 2015). In *functional* neuroimaging studies, researchers are studying how the brain’s activity patterns change during engagement with a particular activity, over time at rest, or when compared across age or disorder status. FMRI measures these activity patterns *via* fluctuations of the brain’s blood oxygenation level over time (Hillman, 2014; Gauthier and Fan, 2019). MRI techniques allow us to collect detailed localization of structure and function down to a few millimeters of the brain’s cortical surface. The eye-catching brain pictures that emerge with an MRI analysis, however, belie the many challenges researchers face when putting together a high-quality developmental cognitive neuroscience experiment.

This review will cover three main topics to help neuroimaging researchers obtain high-quality datasets: (1) recruitment, (2) increasing scan success for researchers *and* participating families, and (3) important considerations when designing a developmentally-appropriate MRI study. In doing so, we refer both to our group’s collective extensive experience using repeated MRI scanning with youth and families, as well as to current best practices in the neuroimaging field. It is important to note that our MRI research study experiences occur in the United States and there may be deviations from our best practices in other countries. Further, we focus this discussion on school-age participants and older, as infant through preschool imaging requires additional techniques and considerations (for working with those under age 6 years, please see, for example, the recent review by Copeland et al., 2021).

## Recruitment techniques

First, we review key recruitment techniques when working with families and youth, including working with non-English speaking families, partnering with school districts, and recruiting children with mental health diagnoses. Developmental research requires family-level and oftentimes community-level research engagement. It is essential that families have positive experiences with the scientific process, and that ethical and procedural issues are settled well in advance of recruitment.

### Engaging representative participants

For much of its brief history, developmental cognitive neuroscience has relied on “convenience” samples, or participants who are often from academic or medical center communities. In the United States, this practice has resulted in recruitment samples that are often primarily white, monolingual, and from families that are affluent and educated (Nketia et al., 2021). As a field, it is critical to diversify our research at all levels, from the science team to the participant pool, in order for our results to generalize to the broader community (for further discussion, please see Garcini et al., 2022). It is more time consuming and challenging to recruit representative and diverse populations for MRI research, but this consideration is essential to reduce bias and measure brain and cognitive development in a generalizable manner. Related to the goal of increasing diversity, researchers need to make it as easy as possible for all types of families to take part. Families should not have to take time off work or miss educational activities in order to participate. One key to offering after school or weekend visit times to families is hiring research staff with flexible schedules, so that the lab can offer research collection visits whenever is best for the families being recruited.

Our lab has used many techniques to recruit participants, including partnering with school districts, online advertising, and community outreach events. Preparing outreach events where we can have face-to-face contact with families alleviates the barrier of being unknown to potential participants. In outreach events, it is helpful to prepare fun, brain-related activities for children to get excited about brain research, and helps to build enthusiasm toward science in general. Through these events, we obtain contact and demographic information and add willing families to a study contact database. Because they have met us, we find these families are often more eager to participate in research studies. Other researchers have found that when working with an underserved population, having a community member that families trust, such as a school staff member (a school counselor), or a priest, endorse the study can motivate families to participate as well (Haack et al., 2012). Purchasing Facebook or other social media ads also provides access to large and diverse samples of a local community (Kosinski et al., 2015).

In our MRI studies of reading intervention and struggling readers with the Texas Center for Learning Disabilities (www.texasldcenter.org), forging school partnerships has been absolutely critical. Hiring school staff to send recruitment materials home, or to make calls to families can greatly improve school-based recruitment. Attending school events, meeting with school administrators, or offering outreach, professional development, or meetings with teachers or students and families, can all help facilitate a strong partnership.

For our studies of children with mental or physical health diagnoses, we have found it useful to advertise with physical/occupational/speech therapy clinics, to visit with large pediatrician practices, and to partner with neuropsychological testing centers. We have also reached out to parent support groups to help with their outreach activities (e.g., fundraising walks/runs) as well as to spread word of our research studies. Being transparent, positive, and willing to adjust and help with the needs of these various groups can help forge lasting, mutually beneficial partnerships.

### Recognizing commitment with compensation

It is common practice to compensate MRI participants for their time and efforts. We offer payments to youth, along with a picture of their brain to take home. Paying the children directly often gives them a sense of ownership and pride over their participation. Parents/guardians should also be compensated for travel expenses (mileage, gas) and their time, especially if a number of parent surveys or other paperwork are requested. Providing families with free reserved parking, giving clear parking directions, and escorting the family from the parking area to and from the imaging center eases stress and saves time. For longitudinal studies, compensation could increase over time, or feature a bonus element for % completion, which motivates families to complete multiple research steps. When possible, research teams should provide families with multiple compensation options such as cash, check, and/or private Venmo transactions. Offering cash as a form of payment is particularly relevant for participant populations who may not have access to a bank account. In addition, or as an alternative to monetary compensation, children may also enjoy selecting a gift such as a toy, book, stuffed animal, or piece of candy from a prize box. Another compensation route may involve providing families with lab-themed items such as water bottles, shirts, or totes, which are both functional and serve as advertisements for the lab.

### Participant retention

Whenever a research study involves multiple visits, there is the risk of attrition, or participants failing to complete all planned visits. This phenomenon may increase as the time gap between research visits increases. Child and adolescent MRI studies often involve multiple visits in order to reduce the length of a single session. In our lab, we typically have a 2–3 h consenting and behavioral data visit (when we also introduce the MRI simulator, see section Recognizing commitment with compensation), and a separate 2–3 h MRI session. We then often follow-up with these families to return 3–6 months or a year later to repeat these activities. There are many approaches to bolster participant retention; we review a few that have been successful for us.

A dedicated website to the project and related elements (directions, scan preparation reminders, recent result updates) can help families feel connected to the project, and even to share the project information with teachers or friends. This website could have recordings of the scanner noise, a video tour of the scanning facility, a timeline of study visits, and more as fits the need of the particular study.

Families who participate in research studies often do so to help advance science, and thus many of our families express an interest in seeing any new findings that have been published as a result of their participation. In addition to posting to a study-related web page, researchers may consider disseminating annual or semi-annual digital or paper newsletters to families which communicate interim findings, describe current lab projects, introduce the research team, and express gratitude for participants. This practice reminds participants that they are integral to our research, and may make them more likely to return for subsequent waves of a longitudinal study. For longitudinal protocols where there is a significant amount of time between waves, it can also be helpful to send reminders either digitally or by mail. Researchers may send birthday or holiday cards to participants and include a gift such as a sticker or small gift card (e.g., Hanna et al., 2014). However, take care to ask families during recruitment if they consent to receiving a holiday or birthday card, as some people may decline due to religious or personal reasons. For longitudinal protocols with a shorter amount of time between waves, it is helpful to schedule the return visit at the initial visit, and the research team should send appointment reminders at regular intervals to allow ample time for families to reschedule their appointments if needed. Regular check-ins to confirm contact information ensures that participant attrition isn’t simply due to a change in address, phone number, or e-mail.

Establishing a welcoming research environment staffed by friendly, experienced, and compassionate researchers also helps to reduce attrition. Throughout the research experience, we provide the participant and family members with parent-approved snacks and water, and we have toys and movies available for any additional family members in the waiting area. Having a free Wi-Fi network and portable desk is also helpful for parents who wish to work during the visit. When staffing permits, it is also beneficial to have a researcher periodically check on the family to provide updates about the participant as well as to ask if they need anything. Alternatively, some of our families have found it helpful for us to provide updates *via* text so that they are kept in the loop about their child’s progress. A participant who feels a sense of trust in the research team is more likely to return for follow-up visits when compared to those who do not have the opportunity to establish rapport with researchers (Young and Dombrowski, 1990; Froelicher et al., 2003). Additionally, they may also be more inclined to tell friends and family about the research, which helps recruitment. After a visit, we assess participant experience through a feedback survey where families have the opportunity to express what they liked or didn’t like about the session and to communicate any suggestions for improvement moving forward. Our team reviews and incorporates this feedback at the end of each collection wave, in the hopes that families have a more positive research experience in the next iteration of data collection. When families withdraw from longitudinal research it is also crucial that researchers ask what led them to stop participating, in order to make improvements to prevent similar experiences moving forward.

Another unique retention strategy that neuroimaging researchers can leverage is to offer a picture of a participant’s brain at the conclusion of a study. We have found that the opportunity for a participant to take home a picture of their brain can be a strong incentive for families who are deciding whether to participate in our research, and it costs virtually nothing for the research team. Our researchers show participants a picture of their brain on the computer at the conclusion of their first scanning visit, and remind them that they will receive a picture of their brain that they can take home when they return for their next (and final) MRI visit. Youth participants often take these pictures to science classes, show them to friends, or post them on social media, which has occasionally served to recruit additional participants for a study.

### Consistency is critical

Consistency across individuals and across lab sessions is a key element of data collection for both data quality and participant experience. Having trained research staff who are experienced at working with MRI and with children is invaluable, but even if a researcher’s prior experience varies, having a similar quality and experience across the duration of study collection should be the focus. Constructing a project recruitment manual for research staff reference promotes consistency of information across different lab members. This manual can include Institutional Review Board (IRB)-approved phrasing of phone call scripts, emails, and text messages (see example phone script in Appendix in Supplementary material). The manual can also help with frequently asked questions (FAQs) by participants and their families. For example, having lab-discussed answers in the manual for explaining what an MRI scan is, how it works (in family-friendly language), that it is non-invasive, and that it does not involve radiation, is important to consistently dispel common misconceptions.

In our recruitment manuals, we include step-by-step guides about the following: accessing contact information, determining eligibility, scheduling time at different facilities, and sending visit confirmation emails (see Appendix in Supplementary material). In our visit confirmation emails, we send families clear directions indicating where the study will take place, and a brief overview of the visit’s activities. One or two days before the scan visit date, we send another reminder email or text asking the family to confirm if they will be able to attend and provide notes about what to wear/not wear. In our experience, this step has vastly reduced the number of “no show” visits. This confirmation step also saves researchers’ time and MRI funds, and opens the opportunity for another participant to be scheduled at that time if the original family cannot attend.

Consistent study information and contact with the family is even more important when working with families whose primary language is not English. Hiring bilingual staff, and using professionally translated recruitment materials, ensures that information is distributed equally to all families and that everyone can have their questions addressed regarding participation. We’ve found that hiring staff who come from the same culture as the families, and speak their language natively, has made the participants feel more comfortable; they have been more open to having conversations, and tend to ask more questions with staff from their cultural and linguistic background.

Having a predictable and consistent environment and messaging is also essential when recruiting children with a mental health diagnosis such as autism spectrum disorder (ASD), anxiety, Tourette disorder, or attention-deficit hyperactivity disorder (ADHD). Having the chance to ask questions in advance, practice the MRI visit (see section Recognizing commitment with compensation), establishing clear expectations and instructions, and having the same researcher present across different research sessions can make all families feel welcome and safe. Research team members should be familiar with the various symptoms that can accompany these disorders, and design a research protocol that allows extra acclimation time and rest breaks if needed.

## Promoting scan success

Next, we discuss ways to reduce scan and participation anxiety for families. It is easy to forget, once researchers themselves are familiar with the MRI environment, how entirely strange and unusual MRI research typically is to youth and their families. If children are familiar with MRI, it may have been through a medical situation that was out of their control. Therefore, it is common for families to experience mild anxiety or even fear to research MRI participation (Westra et al., 2010). Researchers should make the consent process detailed and conversational, especially emphasizing that the participant’s comfort is of priority, and that they are volunteers and can stop participating at any time. Pre-scan anxiety is prompted by a variety of factors, such as being in an unfamiliar medical environment, having to be away from a parent/guardian during the scan, or experiencing feelings of claustrophobia or noise discomfort. There are several things to keep in mind during the design and execution of neuroimaging protocols involving children and adolescents; below, we outline several methods to promote MRI data collection success.

### Pre-screening and MRI contraindications

It is essential to pre-screen potential participants for MRI eligibility and safety well ahead of the MRI appointment date. This approach ensures that any possible contraindications are cleared or attended to ahead of time, and doesn’t waste an ineligible family’s valuable time, or a researcher’s valuable scan hours.

While there are numerous medical and psychological conditions that may make a participant ineligible for a given protocol, the presence of metal orthodontia on the teeth is one of the most common MRI contraindications among child and adolescent research participants. Traditional metal braces and permanent retainers can cause artifacts that distort the image quality, rendering them unusable for analysis (New et al., 1983; Krupa and Bekiesińska-Figatowska, 2015). Dental work is particularly challenging for developmental researchers who conduct longitudinal neuroimaging protocols over the prime orthodontia ages of 8–15 years, because youth may get them on and off over the years of a study. Research teams should ask newly recruited families if and when their child plans to get braces to try to schedule around ineligibility. Newer orthodontic approaches, such as the removable Invisalign aligners, or removable retainers, can sometimes work better for MRI research. As a further way to reduce attrition due to braces, researchers may provide reimbursement for families who choose to pursue these alternative forms of orthodontia. While this is not common, researchers can also partner with specific local orthodontists to expedite the reimbursement process for families. Similarly, the research team can budget for temporary body piercing removal and provide participants with MR-safe plastic piercing retainers. Principal investigators who are interested in providing orthodontia and/or piercing reimbursement for families may budget these expenses into grant proposals.

Other artifact-inducing items that can be more common in child and adolescent groups include metal-infused makeup, glitter in products for the skin or hair, as well as glitter or metal nail polish (Escher and Shellock, 2013). Having a sink to wash skin or hair, and having nail polish and makeup remover in the MRI lab can help prevent unexpected ineligibility on the day of the visit. Another significant concern is the growing number of clothing items being made with silver treated “anti-odor” fibers (Pietryga et al., 2011). Out of an abundance of caution, it is best practice to have MRI participants change into cotton scrubs with no (or sewn shut) pockets. This solution keeps participants safe from metal aspects of clothing, and also keeps children from entering the MRI with coins or other small metal items in their pockets. MRI centers can keep and launder a large variety of sized scrubs for research participants, including child sizes. For those MRI sites without scrubs, researchers should ensure that participants arrive at the imaging center wearing comfortable, MR-safe clothes and that adolescent females wear bras containing no metal components. It is helpful to keep a few MR-safe cotton or polyester sports bras on-hand that female participants can change into if needed.

### MRI simulators and other methods of MRI research preparation

Perhaps the most successful method for preparing MRI-naive youth participants for a research scan is to introduce them to a simulated scanner environment ahead of the scan collection date. Overall scan success among children who participate in a simulated mock scanner environment prior to the real MRI may be higher than those who do not have the opportunity to visit a mock scanner (Hallowell et al., 2008; de Bie et al., 2010; Thieba et al., 2018; Simhal et al., 2021). Other useful aspects of an MRI simulator are to prompt conversation with the participant about how motion corrupts MRI images, and to use the simulator to observe the participant’s ability to hold still. During the simulated mock environment, researchers should communicate the importance of remaining as still as possible while the camera “takes pictures” to avoid any artifacts due to motion. In one study of youth patients with and without ADHD, a mock scanner training protocol which involved providing real-time motion feedback significantly reduced head motion during the real MRI scan. Excessive head motion (>2 mm) in the healthy control group affected 39% of runs during mock scanner training compared to 13% percent of runs once in the real MRI scanner, and excessive head motion affected 51% of runs during mock scanner training in ADHD diagnosed patients, compared to 12% of runs during the actual MRI scan (Epstein et al., 2007).

**FIGURE 1:**
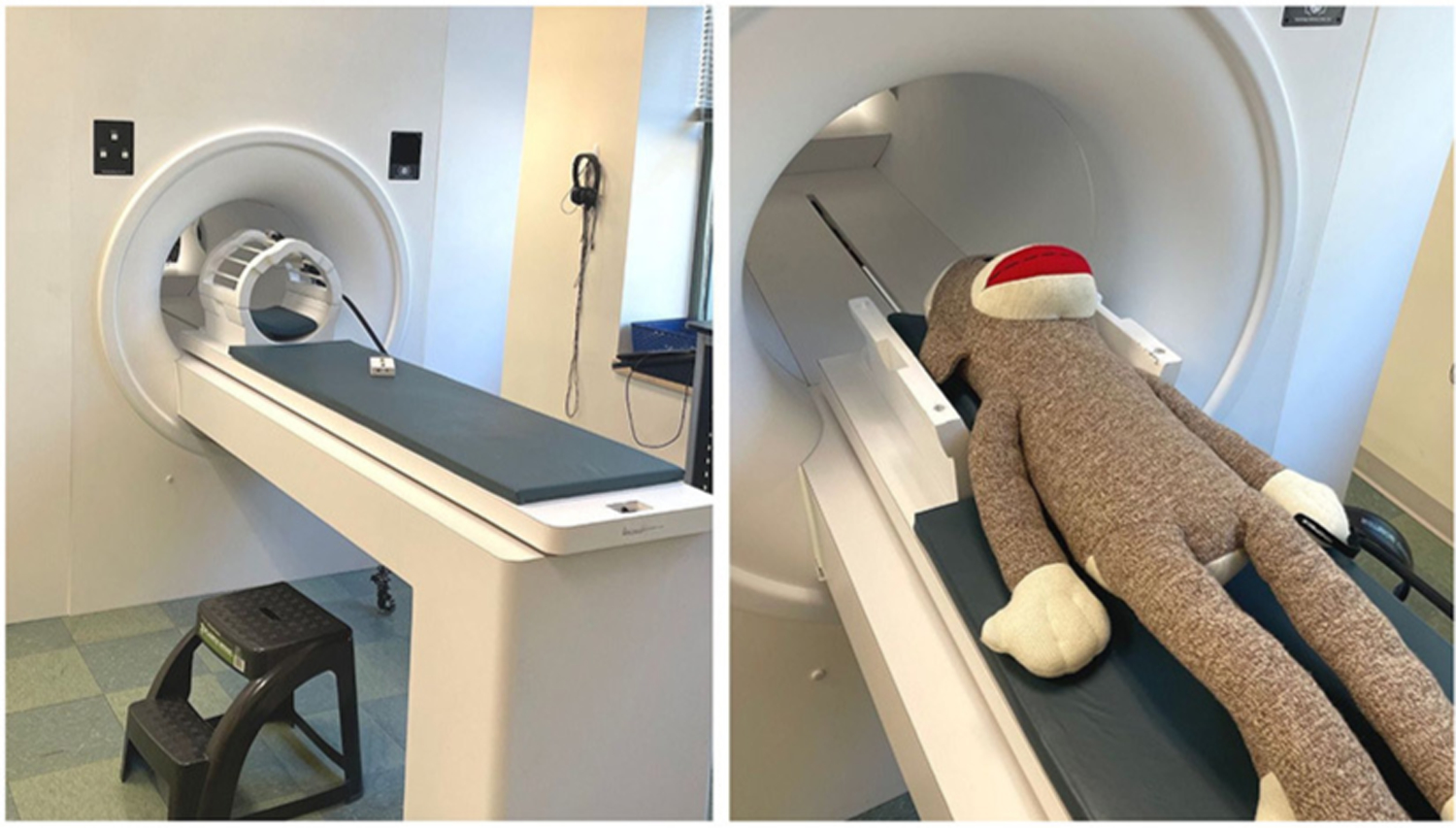
An example of a simple MRI simulator (mock MRI scanner) that is non-magnetic and safe for families to visit and try out. Left: A stepping stool, headphones, head coil, and practice button box are visible. Right: A large stuffed animal (sock monkey) can be used to demonstrate the process for anxious participants or outreach groups.

The purpose of the simulated MRI scanner is to mimic the experience of entering a real MRI and to habituate youth participants to an unfamiliar environment, with the goal of obtaining high quality images during the real MRI scan. Commercial mock scanners have a facade that looks like the “donut-shape” bore of the MRI, a table that rolls into the center of the bore, and a “helmet” that mimics the appearance of a head coil (Figure 1). Mock scanner environments are commonly used with recordings of the MRI auditory environment that mimic the sounds participants will hear during scan sequences. Mock scanners can also include accessories that the participant may encounter during the real MRI, like headphones, a button response box, head pads, or an emergency squeeze ball, which all increase scan environment familiarity. While many large research universities and hospitals invest in a commercially produced mock scanner, there are also low-cost options that successfully mimic the scanner environment using widely accessible materials such as cardboard tubes and wooden tables. One mock scanner environment was created using a children’s play tunnel, a box containing foam padding that resembles head coil, and a massage mat to mimic the vibrations produced by diffusion-weighted imaging (Barnea-Goraly et al., 2013). Alternatively, researchers who do not have the resources to invest in a mock scanner can schedule additional scan time (∼5–10 min) at the beginning of the imaging session to go over important information related to the MRI. This is an opportunity to slowly acclimate the participant to the scanner environment, and to show them how they will be positioned in the scanner before beginning the scan protocol.

Our neuroimaging center provides MRI researchers with a commercial mock MRI scanner produced by Psychology Software Tools which mimics the look and feel of the real MRI scanner. We bring all prospective MRI participants to the mock scanner to gauge their interest and level of comfort ahead of their scan date, usually in combination with obtaining study consent and any neuropsychological and behavioral assessments that are part of our visit protocol. Our researchers describe the purpose of the MRI, what participants can expect during the visit, what the scans sound like, and important safety and comfort considerations related to the MRI visit. Our researchers are sensitive to participants’ emotional responses during the mock scanner experience and we exclude participants who express significant anxiety, sensitivity to noise, demonstrate an inability to briefly hold still, or report claustrophobia. The research team should also be mindful of how a parent/guardian’s presence may pressure or encourage a participant to agree to the MRI. The research team has to use their expertise with scanning this population to determine whether the participant is likely to enjoy the experience and have a chance at being successful during data collection. Regardless of ultimate eligibility or later success, the mock scanner and real scanner visits should always put the volunteer family’s needs and happiness first, and work to make the research experience fun and educational.

### Increasing participant comfort to reduce motion artifact

As noted above, in order to obtain high quality images that can be used for later analysis, it is vital that participants remain as still as possible throughout the course of a scan. Excessive head motion is the *coup de grace* for any neuroimaging protocol, and this issue is particularly relevant in youth samples. Once the child is inside the MRI scanner, time is of the essence. Movement (and chance of the child stopping the protocol early) increases with time (Engelhardt et al., 2017). Fortunately, there are many techniques and tips that can be used to help scan quality during collection, both in the protocol design, and during interactions with participants.

An attentive researcher can help tremendously to quell any anxiety or fears a child may have leading up to the MRI session. It is important to validate a child’s experience of anxiety and ask questions about their expectations of the scan, which can help to alleviate any ambiguity about the visit. We remind participants that their parent/guardian will be nearby, that they can stop participating at any time, and stress that they should tell the researcher if they feel uncomfortable at any point. Our researchers learn to strike a balance between providing gentle encouragement to participants who may feel anxious, while also being mindful when a child appears to be too overwhelmed to continue. Research personnel must also be aware that a child may not be able to accurately communicate their degree of distress due to an implicit pressure to comply with the researchers, fear of disappointing oneself or their parent/guardian, a desire to receive full compensation, or an inability to express their emotional state verbally (Raschle et al., 2012). Anxiety also causes physical tension in the body, and we have found that taking measured steps to ease participant anxiety ahead of the scan often results in a calmer, more relaxed participant. Physical tension is characterized by body rigidity and is regularly accompanied by a desire to move in order to alleviate those feelings of rigidity. Therefore, it is essential that researchers focus on this aspect of participant comfort to prevent any reduction in image quality due to motion artifacts and to ensure participants have an enjoyable research experience.

In addition to addressing anxiety-driven discomfort during a scan visit, there are several physical tools researchers can utilize once in the magnet room to limit participant movement by increasing participant comfort. Restrooms should be visited immediately prior to the scan session. Hair should be unbound, as any (non-metal) hair ties tend to create uncomfortable pressure over time. Use of a non-metal weighted blanket, or small sandbags on the lower legs can also help encourage participants not to cross their limbs and to relax under some positive pressure. Well-fitting earplugs combined with headphones reduce the scanner noise and allow participants to hear movies, any auditory task stimuli, and the researchers from the control room. Researchers can model how to correctly insert an earplug for older participants, and assist younger children (with their consent) to place the earplugs in their ears for maximum efficacy. Once on the MRI table, researchers should ensure the participant’s head is positioned properly in the base of the head coil. There are numerous strategies to restrain the head, from foam cushions and/or inflatable positioning pads, to individualized foam head cases (e.g., Power et al., 2019; Jolly et al., 2020). We give participants ample opportunity to express any points of physical discomfort, and ask if there are ways we can improve their comfort level before beginning the scan session. Participants who are physically uncomfortable may ask to take a break in the middle of a sequence, which consumes valuable scan time and can result in data loss and ultimately ending the scan early.

During the scan session itself, we find that playing a movie for the participant during the structural MRI sequences is particularly helpful with alleviating motion and stress, even in adult participants. Playing the movie again during scanner adjustments (e.g., fieldmaps and shimming) can also provide participants a little break from cognitively demanding activities. Some children can have difficulties transitioning between the movie and research tasks, so set expectations up front about how long each “set of pictures” will be, and consider alternating between research tasks and movie segments. Further, using a movie or offering live motion feedback is shown to result in lower movement for young children in particular (Greene et al., 2018).

Another key strategy has been to keep individual scan sequences engaging by using fun stimuli if possible, and making them as video game-like as possible. Task explanation and practice *prior* to the participant going into the scanner, when the researcher and participant can be face-to-face saves time and increases understanding relative to explaining the tasks over the microphone while the child is in the scanner. However, reminding the participant of instructions and offering them practice with the response device (often a button box) is also helpful immediately prior to the MRI collection of any given task.

Scan operators should check in with participants over the microphone after the conclusion of each scan sequence to ask how they’re doing and to remind them to stay as still as possible before progressing to the next sequence. Researchers who provided pre-scan information related to the MRI, and regularly communicated with participants between scan sequences through the intercom, had participants who experienced significantly lower anxiety levels as indicated by a behavioral inventory and blood cortisol levels, compared with control group participants who received no information or communication (Tazegul et al., 2015). It is important to ask the participant how they’re doing after each scan in positive, child-friendly language, because it gives them explicit permission to communicate any distress or concern before it becomes overwhelming (Raschle et al., 2012). A child’s concept of time is different from an adult’s, so being transparent about how long each scan sequence will take or when they will get to watch the movie next can also help to preserve motivation and reduce anxiety (Qu et al., 2021).

## Key research design considerations when studying development

In this final section, we briefly note certain additional research considerations that are important when designing a study in developmental cognitive neuroscience. All are worth further reading in the developmental cognitive neuroscience literature, as the decision points are complex and require extended consideration (e.g., Luna et al., 2010; Power et al., 2014; Vijayakumar et al., 2018). Careful research design can help maximize the success of developmentally appropriate research questions, and increase the likelihood that researchers are able to draw meaningful conclusions from their datasets. Further, developmental cognitive neuroscience researchers must always remember that the dataset being analyzed is from the (potentially restricted and biased) subset of the population that could access and tolerate the complex MRI research protocol, and always be seeking ways to create protocols where more children and adolescents can be successful.

### Protocol design aspects that can reduce or assess motion artifact

A recent exciting advance is the use of multi-echo fMRI sequences as a denoising tool. These sequences acquire multiple echo images per slice, allowing cleaner separation of the BOLD signal from artifact (e.g., Kundu et al., 2017; Power et al., 2018; Gilmore et al., 2022). This approach often slows repetition times (TRs) of the images a little, but other parameters can be adjusted to compensate. As this is not an element that can be added *post-hoc*, this type of data collection is worth considering prior to beginning an experiment. Using multi-echo sequences can significantly improve signal to noise estimates and may be more sensitive to activity patterns that are lost in noise during single-echo collections (Gilmore et al., 2022).

Time of day can have a big impact on motion and compliance in the scanner: we have found that participants are better able to remain still during a scan on the weekend, or during school breaks, as opposed to being scanned on a weekday evening after school. Participants who are scanned after a long school day may experience more restlessness, hunger, and fatigue, which manifests in more movement during the scan. Additionally, researchers who collect functional task data may see decreased performance in children who are fatigued after a 6–8 h day of schoolwork.

Because children increase in movement over time in the scanner, it is advisable to collect one sequence of each high-priority data type (e.g., Task A, B, and C) prior to repeating any data types. Collecting all iterations of Task A before all of Task B or Task C may result in less full coverage of Task C across individuals due to fatigue, and also creates a consistent disparity between Task A and Task C across participants. Further, children, and those with disorders, can accumulate more discomforts over time (hunger, thirst, movements, fatigue), and risk losing later data points. Relatedly, a rapid re-entry protocol where the participant can come out to go to the bathroom or to stretch, and then get back in to continue the scan session can also help to preserve motivation and endurance in participants who might otherwise end the visit prematurely due to physical discomfort. A bathroom and/or stretch break may also reduce motion artifacts in children who become restless throughout the course of a scan.

Real-time data monitoring can be a highly useful tool for researchers to know whether they are collecting high-quality data from a participant. A few options currently exist: Framewise Integrated Real-time MRI Monitoring (FIRMM) is a software package for purchase, and AFNI software (free) can also be configured to do this monitoring. These software packages provide valuable moment-to-moment data about a participant’s movement during a scan session that is more accurate and reliable than viewing fMRI data by eye as the scan reconstructs on the MRI console. FIRMM software detects motion by calculating and displaying a framewise displacement score, which is a sum of all head movement from frame to frame (Dosenbach et al., 2017). Researchers who conduct neuroimaging protocols with children and adolescents with ADHD or Tourette disorder may particularly benefit from the use of real-time data monitoring, as these diagnoses can cause significant restlessness and/or involuntary movements which affect image quality. This type of movement information, while not “rescuing” contaminated data, allows researchers to make informed decisions about whether to repeat a scan sequence contaminated by motion, or whether to discontinue the session due to excess motion.

After data collection, there are numerous methods to reduce motion artifacts that can be included in the preprocessing steps for both task and resting-state fMRI data (e.g., Power et al., 2014, 2020; Siegel et al., 2014). While there are also strategies for evaluating the quality of structural (e.g., Monereo-Sánchez et al., 2021) and diffusion images (e.g., He et al., 2021), there are fewer ways to correct for motion artifacts after the acquisition process. High-quality data collection is thus particularly vital for structural MRI, but stringent motion control both during and after collection for all scan types improves true signal and reduces the influence of colored and uncolored noise.

### Task design often requires careful behavioral piloting

The choice of age span, stimuli, and scan sequences all strongly impact the conclusions a research study can draw, and the dynamics or trajectory of the developmental cognitive processes that can be observed. Careful consideration is needed in any experimental design to consider the particular developmental period most appropriate for the question, the feasibility of different techniques for getting high quality data from that population, and the variability of other developmental elements in a given age (e.g., pubertal hormones).

Considerable time in task-based fMRI studies can be spent developing any behavioral tasks to be appropriate for the populations of interest (e.g., struggling reader appropriate, ADHD appropriate, adolescent vs. early elementary student appropriate). Out-of-scanner piloting is needed to develop engaging stimuli, and to identify the optimal presentation timing of those stimuli to allow successful performance across different age groups. Each type of task can have specific developmental aspects to consider. For example, for any word-based stimuli, consider the average age that the words are encountered, and the reading speed and comprehension level of the participants; for any rule-based stimuli, consider the working memory load of different ages. Different reading speed in struggling vs. non-struggling readers, for example, can create large group differences in average response time in a sentence reading task, creating potential confounds when comparing the BOLD signal between those groups. Further, if the task is too hard, struggling readers will get frustrated and possibly stop responding, while if the task is too easy, strong readers will get bored and possibly do the same.

The number of different trial types, and number of trials of each type needed for adequate power to detect differences, is also a frequent consideration in task-based fMRI studies. The BOLD signal is noisy, and often multiple iterations of any particular trial type are needed for statistical comparisons. While studies of young adults with similar tasks can give insights as to the number of trials needed for a given comparison, youth samples can be less consistent and may need more trials and practice. The task comparisons between different groups can also depend on the other condition to which the task of interest is being compared (BOLD signal change is always relative). This comparison condition is often fixation or a lack of overt task (i.e., rest), but sometimes it can be an “easier” task where presumptions are made that the easier task is similarly easy for each age group under study (the “task B” problem, see Church et al., 2010; Greene et al., 2016).

A further task element is whether performance changes continuously over the developmental age range, or whether it goes through some discontinuous changes in understanding. For example, some skills continue to improve (e.g., executive function) throughout development, some skills show early growth and then plateau (e.g., response speed tasks), while some skills aren’t present consistently in a given age sample (i.e., word reading in 5–7 year-olds). One type of task design that can adjust performance dynamically over time to keep participants at a similar level of performance is “staircasing”; this approach has been used most often in inhibition tasks (e.g., Roe et al., 2021). While staircasing is not appropriate for many tasks, considering the influence of task performance on research objectives, and doing careful behavioral piloting prior to MRI scanning is clearly essential for high-quality task fMRI data.

### Large samples are needed for certain questions: Public datasets are transformative

One alternative to making all of these decisions and collecting the data oneself, is to turn to publicly available developmental neuroimaging datasets. Resting-state data, because it does not use a custom task that often varies across labs, is particularly amenable to combination across different collections. Underscoring the importance of larger samples, recent neuroimaging studies of brain-wide associations (BWAS; testing for correlations between a behavior of interest across all possible locations in the brain) in resting-state datasets have found that this type of individual differences analysis cannot be reliably studied in small samples, or indeed in samples less than several hundred or thousand people (e.g., Marek et al., 2022). Because of the challenges in developmental neuroimaging discussed above (i.e., motion corruption and attrition both before and during scanning), pediatric MRI or fMRI studies often report smaller samples than ideal for robust conclusions. While it is often difficult for a lab to collect sufficiently large samples for certain research questions locally, another option for developmental cognitive neuroscience questions that require robust power is to use the growing number of large (hundreds to thousands of participants), publicly available datasets of typical and atypical development.

The number of multi-site, public neuroimaging datasets has grown in recent years and researchers have many to choose from, such as the ABCD study (Casey et al., 2018), Human Connectome Lifespan project (Somerville et al., 2018), ABIDE initiative (Di Martino et al., 2014), IMAGEN study (Mascarell Maričić et al., 2020), or YOUth study (Onland-Moret et al., 2020). These tremendous multi-site imaging efforts allow unprecedented statistical power to ask certain developmental questions, or to identify targets for future tailored studies. Further, other researchers can contribute to the data sharing and open science movement by sharing their own data in order for other scientists to use the data in novel ways, or for combining datasets across investigators (e.g., *via* OpenNeuro.org).

## Conclusions

In this article, we have reviewed some of the major participant recruitment, data collection, and study design elements for those entering the field of developmental cognitive neuroscience to consider. From this discussion, there are at least four key takeaways. First, for our studies to be generalizable, it is critical that participants are a representative sample of the local area whenever possible, and thus recruitment efforts must expand beyond traditional convenience sampling approaches. Care and additional community engagement should be employed when approaching and working with vulnerable and underrepresented populations. Second, youth may face burdens to participation that need to be considered and alleviated by the research team, including the form of compensation and transportation support. Consistency during recruitment and collection is key, especially when working with those with mental health difficulties, and longitudinal studies of youth over time also require special considerations to reduce attrition. Third, all MRI research participants can potentially experience heightened anxiety; anxiety can often be reduced with strong research team support, careful study design, and advanced planning. Fourth, and finally, confounds from motion and task performance are considerable difficulties in pediatric samples that can be addressed from a number of different directions to improve data quality.

Non-invasive, minimal-risk neuroimaging techniques like MRI allow unprecedented windows into developing brain structure and function. Developmental cognitive neuroscience research can inform our understanding of how different cognitive or biological trajectories over development can impact real-world outcomes. However, for neuroimaging studies of developmental populations to be successful, there are key considerations related to pediatric-relevant protocol design, recruitment, retention, family and community relationships, data collection, and data analysis.

## Supplementary Material

supplementary material

## Data Availability

The original contributions presented in the study are included in the article/Supplementary material; further inquiries can be directed to the corresponding author.

## Key points from Section 1.

- In order to obtain a representative sample, we have partnered with school districts and community organizations. Hiring a school staff member to do recruitment (someone familiar to families) can build trust and increase participation rates. Other recruitment techniques include social media ads where you can target specific audiences, and hosting outreach events to promote community engagement.
- Establish a welcoming and consistent environment by hiring friendly and experienced researchers who are from similar cultural and/or language backgrounds as the participants.
- To promote positive family experiences and participant retention, it is important to explain the study and processes clearly during recruitment. Offering compensation (e.g., money, travel costs, prizes, a picture of their brain) helps more families to be able to participate.
- Assembling detailed lab manuals and communication scripts facilitates the training of research staff and promotes consistency during data collection (see Appendix in Supplementary material for a brief example).

## Key points from Section 2.

- Prescreen for MRI eligibility during recruitment and again on the day of the scan. We provide safety screening forms to participants to review prior to their visit and follow up with phone calls, especially when following families over time.
- Showing families what the MRI experience is like, *via* a mock scanner or something similar, ahead of the scheduled scan serves to gauge interest and comfort level for families, and reduces unused scanner time expenses.
- Some strategies to keep participants engaged (and still) are: using fun stimuli and short tasks, playing a movie of their choice during structural scans, talking to the participant before and after every scan, and getting participant feedback.

## Key points from Section 3.

- Motion is a common problem in developmental neuroimaging acquisition. Sequence type, time of day, scan order, and participant comfort can differently impact motion and compliance. Researchers can benefit from monitoring motion in real time in order to assess data quality and make acquisition decisions.
- When designing tasks, researchers need to keep the developmental question in mind, as well as statistical power per person and per sample, and age-specific design considerations.
- Public datasets offer increasing opportunities to analyze research questions with more statistical power than individual collections, and at lower cost.

## References

[R1] Barnea-Goraly N, Weinzimer SA, Ruedy KJ, Mauras N, Beck RW, Marzelli MJ, (2013). High success rates of sedation-free brain MRI scanning in young children using simple subject preparation protocols with and without a commercial mock scanner–the diabetes research in children network (DirecNet) experience. Pediatr. Radiol 44, 181–186. doi: 10.1007/s00247-013-2798-7 24096802 PMC3946760

[R2] Casey BJ, Cannonier T, Conley MI, Cohen AO, Barch DM, Heitzeg MM, (2018). The Adolescent Brain Cognitive Development (ABCD) study: imaging acquisition across 21 sites. Dev. Cogn. Neurosci 32, 43–54. doi: 10.1016/j.dcn.2018.03.001 29567376 PMC5999559

[R3] Church JA, Grigorenko EL, and Fletcher JM (2021). The role of neural and genetic processes in learning to read and specific reading disabilities: implications for instruction. Read Res. Q 2021, 1–17. doi: 10.1002/rrq.439 PMC10348696 37456924

[R4] Church JA, Petersen SE, and Schlaggar BL (2010). The “Task B problem” and other considerations in developmental functional neuroimaging. Hum. Brain Mapp 31, 852–862. doi: 10.1002/hbm.21036 20496376 PMC3468298

[R5] Copeland A, Silver E, Korja R, Lehtola SJ, Merisaari H, Saukko E, (2021). Infant and child MRI: a review of scanning procedures. Front. Neurosci 15, 666020. doi: 10.3389/fnins.2021.666020 34321992 PMC8311184

[R6] de Bie HM, Boersma M, Wattjes MP, Adriaanse S, Vermeulen RJ, Oostrom KJ, (2010). Preparing children with a mock scanner training protocol results in high quality structural and functional MRI scans. Eur. J. Pediatr 169, 1079–1085. doi: 10.1007/s00431-010-1181-z 20225122 PMC2908445

[R7] Di Martino A, Yan C-G, Li Q, Denio E, Castellanos FX, Alaerts K, (2014). The autism brain imaging data exchange: towards a large-scale evaluation of the intrinsic brain architecture in Autism. Mol. Psychiatry 19, 659–667. doi: 10.1038/mp.2013.78 23774715 PMC4162310

[R8] Dosenbach NUF, Koller JM, Earl EA, Miranda-Dominguez O, Klein RL, Van AN, (2017). Real-time motion analytics during brain MRI improve data quality and reduce costs. NeuroImage 161, 80–93. doi: 10.1016/j.neuroimage.2017.08.025 28803940 PMC5731481

[R9] Dubois J, Dehaene-Lambertz G, Kulikova S, Poupon C, Hüppi PS, and Hertz-Pannier L (2014). The early development of Brain White matter: a review of imaging studies in fetuses, newborns and infants. Neuroscience 276, 48–71. doi: 10.1016/j.neuroscience.2013.12.044 24378955

[R10] Engelhardt LE, Roe MA, Juranek J, DeMaster D, Harden KP, Tucker-Drob EM, (2017). Children’s head motion during FMRI tasks is heritable and stable over time. Dev. Cogn. Neurosci 25, 58–68. doi: 10.1016/j.dcn.2017.01.011 28223034 PMC5478437

[R11] Epstein JN, Casey BJ, Tonev ST, Davidson M, Reiss AL, Garrett A, (2007). Assessment and prevention of head motion during imaging of patients with attention deficit hyperactivity disorder. Psychiatry Res. Neuroimag 155, 75–82. doi: 10.1016/j.pscychresns.2006.12.009 PMC1993908 17395436

[R12] Escher K, and Shellock FG (2013). Evaluation of MRI artifacts at 3 tesla for 38 commonly used cosmetics. Magn. Reson. Imag 31, 778–782. doi: 10.1016/j.mri.2012.11.002 23290125

[R13] Froelicher ES, Miller NH, Buzaitis A, Pfenninger P, Misuraco A, Jordan S, (2003). The enhancing recovery in coronary heart disease trial (ENRICHD). J. Cardiopulm. Rehabil 23, 269–280. doi: 10.1097/00008483-200307000-00004 12894001

[R14] Garcini LM, Arredondo MM, Berry O, Church JA, Fryberg SA, Thomason ME, (2022, August 17). Increasing diversity in developmental cognitive neuroscience: A roadmap for increasing representation in pediatric neuroimaging research. PsyArXiv [Preprint] doi: 10.31234/osf.io/7ch4w PMC9638728 36335807

[R15] Gauthier CJ, and Fan AP (2019). Bold signal physiology: models and applications. NeuroImage 187, 116–127. doi: 10.1016/j.neuroimage.2018.03.018 29544818

[R16] Gilmore AW, Agron AM, González-Araya EI, Gotts SJ, and Martin A (2022). A comparison of single- and multi-echo processing of functional MRI data during overt autobiographical recall. Front. Neurosci. Brain Imag. Methods 16, 854387. doi: 10.3389/fnins.2022.854387 PMC9081814 35546886

[R17] Greene DJ, Black KJ, and Schlaggar BL (2016). Considerations for MRI study design and implementation in pediatric and clinical populations. Dev. Cogn. Neurosci 18, 101–112. doi: 10.1016/j.dcn.2015.12.005 26754461 PMC4834255

[R18] Greene DJ, Koller JM, Hampton JM, Wesevich V, Van AN, Nguyen AL, (2018). Behavioral interventions for reducing head motion during MRI scans in children. NeuroImage 171, 234–245. doi: 10.1016/j.neuroimage.2018.01.023 29337280 PMC5857466

[R19] Haack LM, Gerdes AC, Cruz B, and Schneider BW (2012). Culturally-modified recruitment strategies for latino families in clinical child research: a critical first step. J. Child Fam. Stud 21, 177–183. doi: 10.1007/s10826-011-9460-5

[R20] Hallowell LM, Stewart SE, de Amorim e Silva CT, and Ditchfield MR (2008). Reviewing the process of preparing children for MRI. Pediatr. Radiol 38, 271–279. doi: 10.1007/s00247-007-0704-x 18084752

[R21] Hanna KM, Scott LL, and Schmidt KK (2014). Retention strategies in longitudinal studies with emerging adults. Clin. Nurse Special 28, 41–45. doi: 10.1097/nur.0000000000000020 PMC3894828 24309576

[R22] He X, Stefan M, Pagliaccio D, Khamash L, Fontaine M, and Marsh R (2021). A quality control pipeline for probabilistic reconstruction of white-matter pathways. J. Neurosci. Methods 353, 109099. doi: 10.1016/j.jneumeth.2021.109099 33582173 PMC8006796

[R23] Hillman EMC (2014). Coupling mechanism and significance of the bold signal: a status report. Annu. Rev. Neurosci 37, 161–181. doi: 10.1146/annurev-neuro-071013-014111 25032494 PMC4147398

[R24] Jernigan TL, Brown TT, Hagler DJ Jr, Akshoomoff N, Bartsch H, Newman E, (2016). The pediatric imaging, neurocognition, and genetics (PING) data repository. NeuroImage 124(Pt B), 1149–1154. doi: 10.1016/j.neuroimage.2015.04.057 25937488 PMC4628902

[R25] Jolly E, Sadhukha S, and Chang LJ (2020). Custom-molded headcases have limited efficacy in reducing head motion during naturalistic fMRI experiments. NeuroImage 222, 117207. doi: 10.1016/j.neuroimage.2020.117207 32745683 PMC7830829

[R26] Kosinski M, Matz SC, Gosling SD, Popov V, and Stillwell D (2015). Facebook as a research tool for the Social Sciences: opportunities, challenges, ethical considerations, and practical guidelines. Am. Psychol 70, 543–556. doi: 10.1037/a0039210 26348336

[R27] Krupa K, and Bekiesińska-Figatowska M (2015). Artifacts in magnetic resonance imaging. Pol. J. Radiol 80, 93–106. doi: 10.12659/pjr.892628 25745524 PMC4340093

[R28] Kundu P, Voon V, Balchandani P, Lombardo MV, Poser BA, and Bandettini PA (2017). Multi-echo fMRI: a review of applications in fMRI denoising and analysis of BOLD signals. Neuroimage 154, 59–80. doi: 10.1016/j.neuroimage.2017.03.033 28363836

[R29] Lerch JP, van der Kouwe AJ, Raznahan A, Paus T, Johansen-Berg H, Miller KL, (2017). Studying neuroanatomy using MRI. Nat. Neurosci 20, 314–326. doi: 10.1038/nn.4501 28230838

[R30] Luna B, Velanova K, and Geier CF (2010). Methodological approaches in developmental neuroimaging studies. Hum. Brain Mapp 6, 863–871. doi: 10.1002/hbm.21073 PMC2907666 20496377

[R31] Marek S, Tervo-Clemmens B, Calabro FJ, Montez DF, Kay BP, Hatoum AS, (2022). Reproducible brain-wide association studies require thousands of individuals. Nature 603, 654–660. doi: 10.1038/s41586-022-04492-9 35296861 PMC8991999

[R32] Mascarell Maričić L, Walter H, Rosenthal A, Ripke S, Quinlan EB, Banaschewski T, (2020). The IMAGEN study: a decade of imaging genetics in adolescents. Mol. Psychiatry 25, 2648–2671. doi: 10.1038/s41380-020-0822-5 32601453 PMC7577859

[R33] Mills KL, Siegmund KD, Tamnes CK, Ferschmann L, Wierenga LM, Bos MGN, (2021). Inter-individual variability in structural brain development from late childhood to young adulthood. Neuroimage 242, 118450. doi: 10.1016/j.neuroimage.2021.118450 34358656 PMC8489572

[R34] Monereo-Sánchez J, de Jong JJA, Drenthen GS, Beran M, Backes WH, Stehouwer CDA, (2021). Quality control strategies for brain MRI segmentation and parcellation: practical approaches and recommendations—insights from the Maastricht study. Neuroimage 237, 118174. doi: 10.1016/j.neuroimage.2021.118174 34000406

[R35] New PF, Rosen BR, Brady TJ, Buonanno FS, Kistler JP, Burt CT, (1983). Potential hazards and artifacts of ferromagnetic and nonferromagnetic surgical and dental materials and devices in nuclear magnetic resonance imaging. Radiology 147, 139–148. doi: 10.1148/radiology.147.1.6828719 6828719

[R36] Nketia J, Amso D, and Brito NH (2021). Towards a more inclusive and equitable developmental cognitive neuroscience. Dev. Cogn. Neurosci 52, 101014. doi: 10.1016/j.dcn.2021.101014 34571453 PMC8476647

[R37] Onland-Moret NC, Buizer-Voskamp JE, Albers MEWA, Brouwer RM, Buimer EEL, Hessels RS, (2020). The Youth Study: rationale, design, and study procedures. Dev. Cogn. Neurosci 46, 100868. doi: 10.1016/j.dcn.2020.100868 33075722 PMC7575850

[R38] Pietryga JA, Fonder MA, Rogg JM, North DL, and Bercovitch LG (2011). Invisible metallic microfiber in clothing presents unrecognized MRI risk for cutaneous burn. Am. J. Neuroradiol 34, 47–50. doi: 10.3174/ajnr.a2827 PMC7964672 22173750

[R39] Power JD, Lynch CJ, Adeyemo B, and Petersen SE (2020). A critical, event-related appraisal of denoising in resting-state fMRI studies. Cereb. Cortex 30, 5544–5559. doi: 10.1093/cercor/bhaa139 32494823

[R40] Power JD, Mitra A, Laumann TO, Snyder AZ, Schlaggar BL, and Petersen SE (2014). Methods to detect, characterize, and remove motion artifact in resting state fMRI. Neuroimage 84, 320–341. doi: 10.1016/j.neuroimage.2013.08.048 23994314 PMC3849338

[R41] Power JD, Plitt M, Gotts SJ, Kundu P, Voon V, Bandettini PA, (2018). Ridding fMRI data of motion-related influences: removal of signals with distinct spatial and physical bases in multiecho data. Proc. Natl. Acad. Sci. USA 115, E2105–E2114. doi: 10.1073/pnas.1720985115 29440410 PMC5834724

[R42] Power JD, Silver BM, Silverman MR, Ajodan EL, Bos DJ, and Jones RM (2019). Customized head molds reduce motion during resting state fMRI scans. NeuroImage 189, 141–149. doi: 10.1016/j.neuroimage.2019.01.016 30639840

[R43] Qiu A, Mori S, and Miller MI (2015). Diffusion tensor imaging for understanding brain development in early life. Annu. Rev. Psychol 66, 853–876. doi: 10.1146/annurev-psych-010814-015340 25559117 PMC4474038

[R44] Qu F, Shi X, Zhang A, and Gu C (2021). Development of young children’s time perception: effect of age and emotional localization. Front. Psychol 12, 688165. doi: 10.3389/fpsyg.2021.688165 34168601 PMC8217659

[R45] Raschle N, Zuk J, Ortiz-Mantilla S, Sliva DD, Franceschi A, Grant PE, (2012). Pediatric neuroimaging in early childhood and infancy: challenges and practical guidelines. Ann. NY Acad. Sci 1252, 43–50. doi: 10.1111/j.1749-6632.2012.06457.x 22524338 PMC3499030

[R46] Roe MA, Engelhardt LE, Nugiel T, Harden KP, Tucker-Drob EM, and Church JA (2021). Error-signaling in the developing brain. Neuroimage 227, 117621. doi: 10.1016/j.neuroimage.2020.117621 33301938 PMC7977480

[R47] Sherman LE, Payton AA, Hernandez LM, Greenfield PM, and Dapretto M (2016). The power of the like in adolescence: effects of peer influence on neural and behavioral responses to social media. Psychol. Sci 27, 1027–1035. doi: 10.1177/0956797616645673 27247125 PMC5387999

[R48] Siegel JS, Power JD, Dubis JW, Vogel AC, Church JA, Schlaggar BL, (2014). Statistical improvements in functional magnetic resonance imaging analyses produced by censoring high-motion data points. Hum Brain Mapp 35, 1981–1996. doi: 10.1002/hbm.22307 23861343 PMC3895106

[R49] Simhal AK, Filho J, Segura P, Cloud J, Petkova E, Gallagher R, (2021). Predicting multiscan MRI outcomes in children with neurodevelopmental conditions following MRI simulator training. Dev. Cogn. Neurosci 52, 101009. doi: 10.1016/j.dcn.2021.101009 34649041 PMC8517836

[R50] Somerville LH, Bookheimer SY, Buckner RL, Burgess GC, Curtiss SW, Dapretto M, (2018). The Lifespan Human Connectome Project in Development: a large-scale study of brain connectivity development in 5–21 year olds. NeuroImage 183, 456–468. doi: 10.1016/j.neuroimage.2018.08.050 30142446 PMC6416053

[R51] Tazegul G, Etcioglu E, Yildiz F, Yildiz R, and Tuney D (2015). Can MRI related patient anxiety be prevented? Magn. Reson. Imag 33, 180–183. doi: 10.1016/j.mri.2014.08.024 25172986

[R52] Thieba C, Frayne A, Walton M, Mah A, Benischek A, Dewey D, (2018). Factors associated with successful MRI scanning in unsedated young children. Front. Pediatr 6, 146. doi: 10.3389/fped.2018.00146 29872649 PMC5972312

[R53] Turk E, van den Heuvel MI, Benders MJ, de Heus R, Franx A, Manning JH, (2019). Functional connectome of the fetal brain. J. Neurosci 39, 9716–9724. doi: 10.1523/jneurosci.2891-18.2019 31685648 PMC6891066

[R54] Vijayakumar N, Mills KL, Alexander-Bloch A, Tamnes CK, and Whittle S (2018). Structural brain development: a review of methodological approaches and best practices. Dev. Cogn. Neurosci 33, 129–148. doi: 10.1016/j.dcn.2017.11.008 29221915 PMC5963981

[R55] Westra AE, Zegers MP, Sukhai RN, Kaptein AA, Holscher HC, Ballieux BE, (2010). Discomfort in children undergoing unsedated MRI. Eur. J. Pediatr 170, 771–777. doi: 10.1007/s00431-010-1351-z 21120526 PMC3099003

[R56] Young CL, and Dombrowski M (1990). Psychosocial influences on research subject recruitment, enrollment and retention. Social Work Health Care 14, 43–57. doi: 10.1300/j010v14n02_04 2631281

