# Supplementary material for: Key considerations for child and adolescent MRI data collection
Source: Front Neuroimaging. 2022 Sep 12;1:981947. doi: 10.3389/fnimg.2022.981947 (PMC9615104; doi:10.3389/fnimg.2022.981947)
Supplement: Supplementary file 1 [file Presentation_1.pdf]

APPENDIX: Example phone recruitment script

All participant communications should be IRB approved. While a version of this script is approved by our IRB for our specific project, this example is not to be used without seeking your own IRB approval.

****

Note: Have the following open and ready to go: Project Recruitment sheet, Lab Google Calendar, Recruitment checklist

REDCap Developmental Pre-Screening Survey (for MRI calls)

****USE GOOGLE VOICE NUMBER****

Recruitment Voicemail:

"Hi, this is _________ from the X Lab at X University. I'm calling you because we are recruiting 4th and 7th grade kids for a new study to come in to the lab [in month] and [longitudinal time, e.g., 3 months]. You'd come in for 2-4 visits, during which your child: (child's name) *would play computer games, do some paper tests and complete an MRI scan if they're eligible.* (Child's name) *would earn $X to $X per visit and take home a picture of their brain, and you would earn $X per visit. If this sounds like something you'd be interested in, please give us a call back at XXX-XXX-XXXX or you can respond to the email we sent you. Thank you!"*

If they pick up the call:

Hi I'm ________ from the X Lab at X University. May I speak with (parent's name)*? Hi, I'm calling you because your family previously signed up to participate in research at X University, does that ring a bell?*

Great, I'm calling you because we want to invite you and (child's name) *to participate in a research study that looks at brain development in kids. This research is being led by Dr. X, who is a professor at X university, and we are looking for kids finishing up 4th or 7th grade to participate.*

Do you have about 10 minutes to learn more about our study?

No - *Are there any concerns you have that we could address? Thank you for your time.*

Yes -
Great! Is your child in 4th or 7th grade?

(Also ask if child has any siblings in 4th or 7th grade)

If NO: *Right now we are only recruiting fourth and seventh graders. Is it OK if we reach out to you in ____(whenever they'll be in 4th/7th grade)____ to see if you're still interested?*

If YES: *OK, now let me take a minute to explain the basics of the study and then I'll answer any questions you might have, does that sound OK?*

So, if (child's name) *is eligible, we would like for you to come to our lab on the X University campus for 2 to 4 visits. The first two visits would take place in [months]. The first visit is behavioral; we would have your child play some computer and paper games and tests, complete questionnaires, and visit a pretend MRI machine.* ***You*** *will also complete some questionnaires during the session. We follow COVID protocols to ensure the safety of your family and our researchers throughout the session as well.*

If (child's name) *is interested and eligible, we will also invite you to come in for a second MRI visit about a week after the behavioral visit. For the MRI visit, we would have* (child's name) *watch a movie and play some games inside an MRI scanner. Your child will repeat these same visits in [longitudinal time, e.g., 3 months]. Does that sound like something you and* (child's name) *might be interested in?*

Do you have any experience with MRIs?

- If NO/minimal experience: *The MRI is essentially a big magnet that takes pictures of your brain and most importantly does not use radiation. There's nothing invasive and it doesn't hurt. We'll take pictures of* (child's name) *brain while they play games and watch a part of a movie so we can see what parts of the brain they are using at different times. We try to make the experience as comfortable and fun as possible: We give* (child's name) *headphones and a microphone to keep an open line of communication, blankets to keep them warm, and foam padding to keep them comfortable. We talk to them in between each set of pictures to see how they are doing.*

Does that seem like something _____ might be OK with doing?

Compensation

- (child's name) *will get paid $X for the first behavioral visit, and $X for each MRI visit, and they will receive a picture of their brain if they participate in the MRI part of the study. You will also be paid $X/visit for driving and completing questionnaires and surveys during visit 1.*
- *Participation in the study is voluntary, so* (child's name) *can quit at any time. However, we are very excited for them to participate and help us out with our research*
- *We can schedule visits during the week after-school or on weekends. We provide you with directions and free parking near our lab. The first behavioral visit takes about 3 hours and the second MRI visit will take about 2.5 hours.*

How does all of that sound?

NO - *Are there any concerns you have that we can address? Well, thank you for your time.*

YES - *Awesome, do I have your authorization to collect some personal information that will be used for research purposes only? Your responses will be kept confidential and we do not share them with anyone outside of our research team.*

*****OPEN UP REDCAP IRC SCREENING FORM*****
PRESCREENING Qs:
Is your child in 4th or 7th grade?
Does your child have any medical, genetic, or psychiatric conditions?
Does your child have braces?

If YES: *"Can you share with me their condition(s)?"*
Usually, parents don't need to be prompted, they'll let you know their child's diagnosis(es) when you ask the first Q

***If at this point they're still eligible, go through the entire REDCap Developmental Subject screening survey with them to determine MRI eligibility. ***
